# Growth of the Obligate Anaerobe *Desulfovibrio vulgaris* Hildenborough under Continuous Low Oxygen Concentration Sparging: Impact of the Membrane-Bound Oxygen Reductases

**DOI:** 10.1371/journal.pone.0123455

**Published:** 2015-04-02

**Authors:** Fanny Ramel, Gael Brasseur, Laetitia Pieulle, Odile Valette, Agnès Hirschler-Réa, Marie Laure Fardeau, Alain Dolla

**Affiliations:** 1 Aix-Marseille Université, CNRS, LCB-UMR7283, Marseille, France; 2 Aix-Marseille Université, Université de Toulon, CNRS, IRD, MIO, UM110, 13288 Marseille, Cedex 09, France; CEA-Saclay, FRANCE

## Abstract

Although obligate anaerobe, the sulfate-reducing bacterium *Desulfovibrio vulgaris* Hildenborough (*Dv*H) exhibits high aerotolerance that involves several enzymatic systems, including two membrane-bound oxygen reductases, a bd-quinol oxidase and a cc(b/o)o_3_ cytochrome oxidase. Effect of constant low oxygen concentration on growth and morphology of the wild-type, single (*Δbd*, *Δcox*) and double deletion (*Δcoxbd*) mutant strains of the genes encoding these oxygen reductases was studied. When both wild-type and deletion mutant strains were cultured in lactate/sulfate medium under constant 0.02% O_2_ sparging, they were able to grow but the final biomasses and the growth yield were lower than that obtained under anaerobic conditions. At the end of the growth, lactate was not completely consumed and when conditions were then switched to anaerobic, growth resumed. Time-lapse microscopy revealed that a large majority of the cells were then able to divide (over 97%) but the time to recover a complete division event was longer for single deletion mutant *Δbd* than for the three other strains. Determination of the molar growth yields on lactate suggested that a part of the energy gained from lactate oxidation was derived toward cells protection/repairing against oxidative conditions rather than biosynthesis, and that this part was higher in the single deletion mutant *Δbd* and, to a lesser extent, *Δcox* strains. Our data show that when *Dv*H encounters oxidative conditions, it is able to stop growing and to rapidly resume growing when conditions are switched to anaerobic, suggesting that it enters active dormancy sate under oxidative conditions. We propose that the pyruvate-ferredoxin oxidoreductase (PFOR) plays a central role in this phenomenon by reversibly switching from an oxidative-sensitive fully active state to an oxidative-insensitive inactive state. The oxygen reductases, and especially the bd-quinol oxidase, would have a crucial function by maintaining reducing conditions that permit PFOR to stay in its active state.

## Introduction

Sulfate-reducing bacteria (SRB) are anaerobic microorganisms ubiquitously distributed, even in atypical environments for this physiological group, e.g. in aerobic layer of a stratified fjord [1], in aerobic wastewater biofilms [2–3], in oxic layers of microbial mats [4–8] or in oxic marine sediment layers close to the sediment surface [9]. Microscopy of roots and rhizomes also revealed the presence of SRB in the sea grass rhizosphere sediments [10], on the surfaces [11], inside epidermal and exodermal cells [12], even deep into the cortex cells of aquatic plants roots [13]. All these ecological niches can temporary be exposed to oxygen concentration up to saturation [4,14–15] and thus force SRB to cope with elevated oxygen tension.

Although SRB are classified as strict anaerobes [16], these examples above suggest that they exhibit high aerotolerance capabilities. Numerous laboratory works have tried to evaluate this aerotolerance, by studying impact of temporary oxygen stresses on various sulfate-reducing bacteria species. The effects of oxygen are visible at either protein [17], transcript [18–21], metabolite [22–24] and morphology levels [25] even at low concentration (0.1% O_2_) or short-term exposure. But SRB appear not to be killed by a simple contact with oxygen and are able to cope with oxygen for several hours or even days in pure culture [26–29]. Together with ecological data [8,30–31], these studies reveal an aerotolerance variability in the various strains of SRB tested, with a higher oxygen tolerance for the members of the genus *Desulfovibrio* [28]. Several studies have even suggested a possible advantage of oxygenic conditions. Jonhson et *al*. [32] succeeded to observe the formation of a focused band of *Desulfovibrio vulgaris* Hildenborough (*Dv*H) cells in an oxygen gradient, at an O_2_ concentration between 0.02 and 0.04% O_2_. Other artificial oxygen gradient experiments revealed growth of SRB close to the oxic-anoxic interface [27,33]. These data point out a positive aerotaxis that would enable bacteria to find environmental conditions favourable for their metabolic lifestyle. In homogeneously aerated pure cultures of several sulfate-reducing bacteria, it has been shown that rate of sulfide formation from the reduction of either sulfate, sulfite or thiosulfate decreased as the oxygen concentration increased, and was abolished above 15 μM [27]. In a lactate/sulfate medium, growth of *Dv*H was shown to be not significantly affected by up to constant 0.04% O_2_ in a N_2_-H_2_-CO_2_ gas mixture sparging while it was inhibited at 0.08% O_2_ [32]. Sigalevich and Cohen [34] reported that when an initially established chemostat coculture of *Desulfovibrio oxyclinae* and the facultative heterotrophic aerobe *Marinobacter sp*. strain MB grown under anaerobic conditions in lactate/sulfate medium was exposed to an oxygen flux, the sulfate reducing bacterium performed an incomplete oxidation of lactate to acetate. The authors suggested thus that in these steady-state continuous culture, *D*. *oxyclinae* was able of oxygen-dependent growth [34]. Even if none of the SRB isolated so far can either grow aerobically or reduce sulfate under high oxygen concentrations, several strains of *Desulfovibrio* species have been demonstrated to have the capability to couple oxygen reduction with proton translocation and energy conservation [35].

The ability for oxygen reduction is widespread among the SRB. The substrates used for this reduction are normally the same as those used for sulfate reduction. So far, H_2_, formate, lactate, ethanol and pyruvate have been shown to be oxidized in the presence of oxygen [36]; however, the highest oxygen uptake rates of several *Desulfovibrio* species were obtained with H_2_ as electron donor [37]. In *Desulfovibrio termitidis*, an oxygen reduction rate of about 1570 nmol of O_2_ min^-1^mg^-1^ of protein was found, higher than the value found in most aerobic bacteria (~700 nmol of O_2_ min^-1^mg^-1^) [38]. It has been proposed that enzymes involved in the energy metabolism during the anaerobic sulfate respiration, i.e., hydrogenases and c-type cytochromes, also are enable of H_2_-dependent oxygen reduction [18,39–40]. In *Desulfovibrio* species, a rubredoxin-oxygen oxidoreductase (ROO) has been shown to be the terminal enzyme of a cytoplasmic NADH-linked non-energy-conserving chain that reduced oxygen in water [41–43]. Wildschut [44] pointed out that O_2_ reduction activity by ROO accounted for 20 to 40% of the total specific oxygen reduction rate of *DvH*. The authors also highlighted the importance of ROO in the survival of the cells to microaerophilic conditions, but not under fully aerobic conditions. Sulfate reducers have also the ability to consume oxygen at the membrane level. Several isolates of *Desulfovibrio* and *Desulfomicrobium* from salt-marsh sediments were found to contain cytochrome bd oxidase and/or cytochrome c oxidase encoding genes [45]. In *D*. *gigas*, a high affinity bd-quinol oxidase was isolated, characterized, and shown to completely reduce oxygen to water [46–47]. In *Dv*H, two membrane-bound terminal oxidases have been characterized, a bd-quinol oxidase and a cytochrome c oxidase that was of a new cc(o/b)o_3_ type, using the monohaem cytochrome c_553_ as electron donor [48]. The *Dv*H *cyd* genes, encoding the bd-quinol oxidase, were 36-fold more transcribed than the *cox* genes, encoding the cc(b/o)o_3_ oxidase, which could be related with the high oxygen reduction rate in the presence of menadiol. Analysis of a *cyd* deletion mutant strain of *Dv*H pointed out the existence of an electronic link between the periplasmic H_2_ oxidation by hydrogenases and the membranous reduction of O_2_ by the *bd-*quinol oxidase [26]. Even if the two membrane terminal oxidases appeared involved in the survival of *Dv*H under low (0.1%) and saturated oxygen conditions, the *cox deletion mutant* was slightly more sensitive, pointing out the importance of the *cc(b/o)o*_*3*_ cytochrome oxidase in oxygen protection [26]. In addition to the O_2_-reduction capability, *Desulfovibrio* strains have developed other strategies to cope with oxygen and protect enzymes from oxidative damages. One of them is a specific reversible thiol-disulfide redox switch, efficient in the pyruvate-ferredoxin oxidoreductase (PFOR) that catalyses the oxidative decarboxylation of pyruvate forming acetyl-coenzyme A [49]. This mechanism involves the reversible formation of a disulfide bond in the C-terminal domain of the PFOR. During the oxidative time period, PFOR switches to an inactive but O_2_-stable form triggered by the formation of an intramolecular disulfide bridge, this specific conformation allowing the protection of a [4Fe-4S] cluster from oxidative damages [24]. Once conditions return to reductive, the disulfide bond is reduced by using a thioredoxin/thioredoxin reductase system [50] and the PFOR is fully reactivated, leading to the restoration of a high rate of pyruvate oxidation in the cells without *de novo* synthesis of this key enzyme. This mechanism is highly valuable for an enzyme that catalyses a crucial step in carbon and energy metabolism of SRB.

As mentioned above, most of the aerotolerance capabilities description of *Desulfovibrio* strains are from temporarily oxygen exposed cells studies. In the present study, the effect of a continuous low oxygen concentration gas mixture sparging on growth, morphology, metabolic activities and cell viability of *Desulfovibrio vulgaris* Hildenborough is reported. In addition, the importance of each of the membrane bound oxygen reductase under these growth conditions is described.

## Materials and Methods

### Bacterial growth conditions

*Desulfovibrio vulgaris* Hildenborough (*Dv*H) was grown at 33°C in liquid lactate (37mM)/sulfate (32mM) medium (medium C) under anaerobic conditions in 10 ml Hungate tubes, inoculated at 10% (vol/vol) as previously described [16]. It should be noted that in this medium, lactate is the limiting factor for growth since two lactate molecules are required to reduce one sulfate in H_2_S [16]. A system using a gas mixer (PEGAS 4000 MF) was developed for a constant gas sparging of the cultures in Hungate tubes with very low concentration of oxygen (from 0% to 0.1%) and high accuracy. In this case, cultures in Hungate tubes (10 ml medium C) were inoculated at 15% (vol/vol) with a pre-culture at OD_600_ ~0.6, and continuously sparged with 0.02% O_2_/ 99.98% N_2_ gas mix. Dissolved oxygen concentration was monitored by using a Mettler-Toledo M700 recorder equipped with an O_2_ module ppb 4700 and a calibrated Inpro 6900 O_2_ probe. Single *Dv*H deletion mutant strains of the genes encoding the bd-quinol oxidase (*Δbd*) and the cytochrome cc(o/b)o_3_ oxidase encoding genes (*Δcox*) as well as the double deletions mutant strain (*Δcoxbd*) [26] were cultured under the same conditions as the wild-type strain. Growth resumption studies after constant 0.02% O_2_ gas mixture sparging were achieved by bubbling the cultures for 25 min with 100% N_2_ to remove traces of oxygen and then incubating the closed Hungate tubes for 20 hours more at 33°C. Growth was monitored by following the optical density at 600 nm. Growth rates were estimated by fitting the scatter plots of logarithmic optical density at 600nm with a linear regression from five independent experiments.

To determine the number of cells per volume unit of culture media, an aliquot of each culture was sampled and placed on a Thoma cell counting chamber (0.0025 mm^2^–0.01 mm depth, Prolabo). At least 20 squares were observed to have a robust measure of the number of bacteria per ml of culture. A correlation factor between the optical density at 600 nm and the number of cells in cultures was determined under both anaerobic and constant 0.02% O_2_ gas mixture sparging conditions.

### Organic acids quantification

Lactate and acetate were quantified by high-performance liquid chromatography (HPLC). Samples of cultures (500 μL) were collected and centrifuged 10 min at 10000*g*. The supernatants were analysed by high-performance liquid chromatography using a SpectraSERIES P100 pump equipped with a SpectraSYSTEM RI-150 detector and an Aminex HPX-87H-300x7.8 mm column C18 (Bio-Rad). Column temperature was 35°C and eluant (H_2_SO_4_, 0.005N) was used at a flow rate of 0.6 mL/min. 20 μl supernatant was injected.

### Microscopy analyses

Microscopy analyses were performed on a temperature-controlled (33°C) TE2000-E-PFS inverted epifluorescence microscope (Nikon, France). Images were recorded with a CoolSNAP HQ2 (Roper Scientific, Roper Scientific SARL, France) and a 100x/1.4 DLL objective coupled with a camera (Hamamatsu Orca-R2). Images were viewed using NIS-Elements Viewer 4.0. The impact of oxidative conditions on cell morphology was determined by measuring the length of at least 300 cells for each strain and condition (anaerobic/oxidative) from two independent experiments using the software Fiji [51]. For cell division analysis by time-lapse microscopy, cells were cultured with constant 0.02% O_2_ gas mixture sparging until they stopped to grow (about 25 h) and conditions were switched to anaerobic by bubbling with N_2_ for 25 min to remove traces of oxygen from the medium. In an anaerobic Coy chamber (5% H_2_, 95% N_2_ gas atmosphere), 3μL of the culture was then placed between the coverslip and a thin layer of medium C supplemented with 1.5% of Phytagel in an hermetic chamber and sealed (Fievet, unpublished). This observation chamber was then transferred to the TE2000-E-PFS microscope. Phase-contrast images were acquired every 10 min for at least 16 hours. The number of cells able to divide, times to recover a complete division event and second division times were determined manually.

### PFOR activity assays

Pyruvate-Ferredoxin Oxidoreductase (PFOR) activity was determined spectrophotometrically at 30°C by measuring the reduction of methyl viologen as previously described [49]. Briefly, the reaction mixture containing 50 mM Tris-HCl (pH 8.5), 10 mM sodium pyruvate, 0.1 mM sodium coenzyme A and 2 mM methyl viologen, was bubbled with argon for 25 min. Then, 10 μl Triton X100 (1/10 in Tris-HCl buffer, pH 8.5), kept under anaerobic conditions in an anaerobic chamber (Jacomex BS531NMT), was added and the reaction was started by injection of 50 μl of cells culture into the cuvette using a gastight syringe. Absorbance at 604 nm was monitored and when variation of absorbance over time appeared linear, 15mM dithioerythritol (DTE) was added anaerobically into the cuvette to reach the maximum of activity which corresponded to a totally reactivated enzyme. The rate of inactive and thus protected enzyme was calculated by the difference of the slope before and after the addition of DTE, reported to the optical density at 600 nm of the culture. As PFOR in cells cultured under anaerobic conditions is fully active [24], the corresponding value of the PFOR protected rate for the wild-type strain was arbitrary set to 1 and all other values were related to this unit.

## Results

### Effect of constant low oxygen concentration sparging on growth

**Fig 1:**
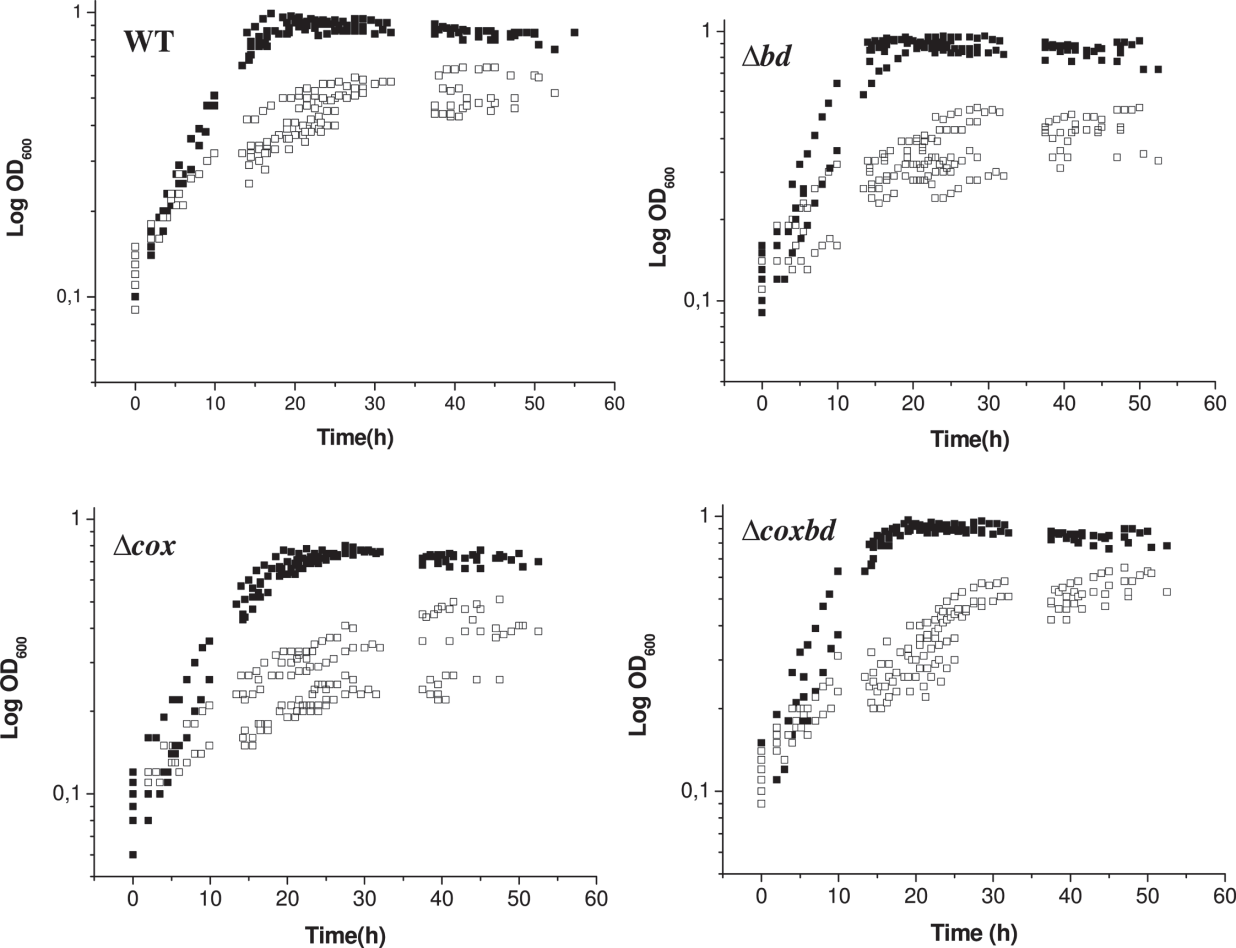
Growth analysis of the *Dv*H strains. Growth curves of the wild-type and the three deletion mutant strains under anaerobic conditions (black symbols) or with constant 0.02% O_2_ gas mixture sparging (open symbols). Data arose from five independent growth experiments.

Wild-type *Dv*H and both single (*Δbd*, *Δcox*) and double (*Δcoxbd*) mutants were cultured in lactate/sulfate medium in Hungate tubes with constant sparging with 0.02% O_2_ in order to evaluate the role of the membrane bound oxygen reductases in the growth under these oxidative conditions. The 0.02% O_2_ sparging conditions led to a constant dissolved oxygen concentration of 0.23 μM in the tubes as measured with the Mettler-Toledo O_2_ probe. Fig 1 shows that all strains were able to grow (about 2.5, 1.8, 2 and 2.4 division events for the wild-type, *Δcox*, *Δbd *and *Δcoxbd* strains, respectively). The oxygen concentration of 0.02% was determined as the highest that permitted growth of both the wild-type and the deletion mutants strains. The growth parameters, determined from the growth curves, are shown in Table 1. Under anaerobic conditions, the doubling time of the wild-type strain was found in the same range (about 5 h) as that of the deletion mutant strains, except for the *Δcox* strain which exhibited a slightly increased doubling time (5.97 h). In the same way, the final biomass was similar for all the strains, except for the *Δcox* strain which showed a lower final biomass (0.45 *vs* 0.55 10^9^ cells ml^-1^). When cultured with constant 0.02% O_2_ gas mixture sparging, the doubling time of the wild-type strain largely increased to around 14 h, and the final biomass was found much lower than under anaerobiosis, 0.29 *vs* 0.55 10^9^ cells ml^-1^. Under these oxidative conditions, the single deletion *Δcox* and *Δbd* mutant strains exhibited also higher doubling times and lower final biomasses than under anaerobic conditions (Table 1). In the case of the *Δcoxbd* strain, the shape of growth curves was quite atypical when cultured with constant 0.02% O_2_ sparging, probably with a biphasic behaviour, preventing the calculation of relevant doubling time (Fig 1). However, the growth curves suggested that the doubling time of the double mutant strain was lower than that of the wild-type under constant 0.02% O_2_ gas mixture sparging. While the *Δbd* and *Δcox* strains exhibited a significant lower final biomass than the wild-type when cultured with constant 0.02% O_2_ sparging, the *Δcoxbd* strain had, surprisingly, a final biomass similar as that of the wild-type strain. These data show that under the oxidative conditions caused by the constant 0.02% O_2_ gas mixture sparging, cells were able to divide; however, less amount of cells were obtained compared to anaerobic conditions, indicating a growth arrest. It should be noted that culturing the wild-type strain with constant 0.02% O_2_ gas mixture sparging did not induce any significant variation in the amount of the *cox* and *bd* transcripts as quantified by qRT-PCR, compared to anaerobic growth conditions (S1 Fig.). In addition, no transcriptional compensation mechanism was found in any of the single deletion mutant strains; a decrease (2.5 times) in the amount of *cox* transcript was however quantified in the *Δbd* strain (S1 Fig.).

**Table 1:** Growth parameters for the *Desulfovibrio vulgaris* Hildenborough strains.

****Genotype****	****Condition****	****Growth rate μ (h**** ^-1^ ****)****	****Doubling time (h)****	****Final biomass (x10**** ^9^ ****cells.ml**** ^-1^ ****)****
WT	anaerobic	0.13	5.21	0.55 ± 0.03 ^a^
	0.02% O_2_	0.05	14.28	0.29 ± 0.03 ^b^ ^,^ ^c^
*Δbd*	anaerobic	0.13	5.33	0.54 ± 0.02
	0.02% O_2_	0.04	15.61	0.22 ± 0.03 ^b^
*Δcox*	anaerobic	0.12	5.97	0.45 ± 0.02 ^a^
	0.02% O_2_	0.04	15.53	0.19 ± 0.04 ^c^
*Δcoxbd*	anaerobic	0.13	5.33	0.55 ± 0.03
	0.02% O_2_	nd	nd	0.27 ± 0.03

*Dv*H strains were grown in lactate/sulfate medium under anaerobic conditions or under constant 0.02% O_2_ gas mixture sparging. Data are mean values of five independent experiments. Statistical analysis using t-test was used to compare means.

^a, b, c^: Significant differences (p<0.05). nd: not determined.

### Effect of oxygen on the cell morphology

**Fig 2:**
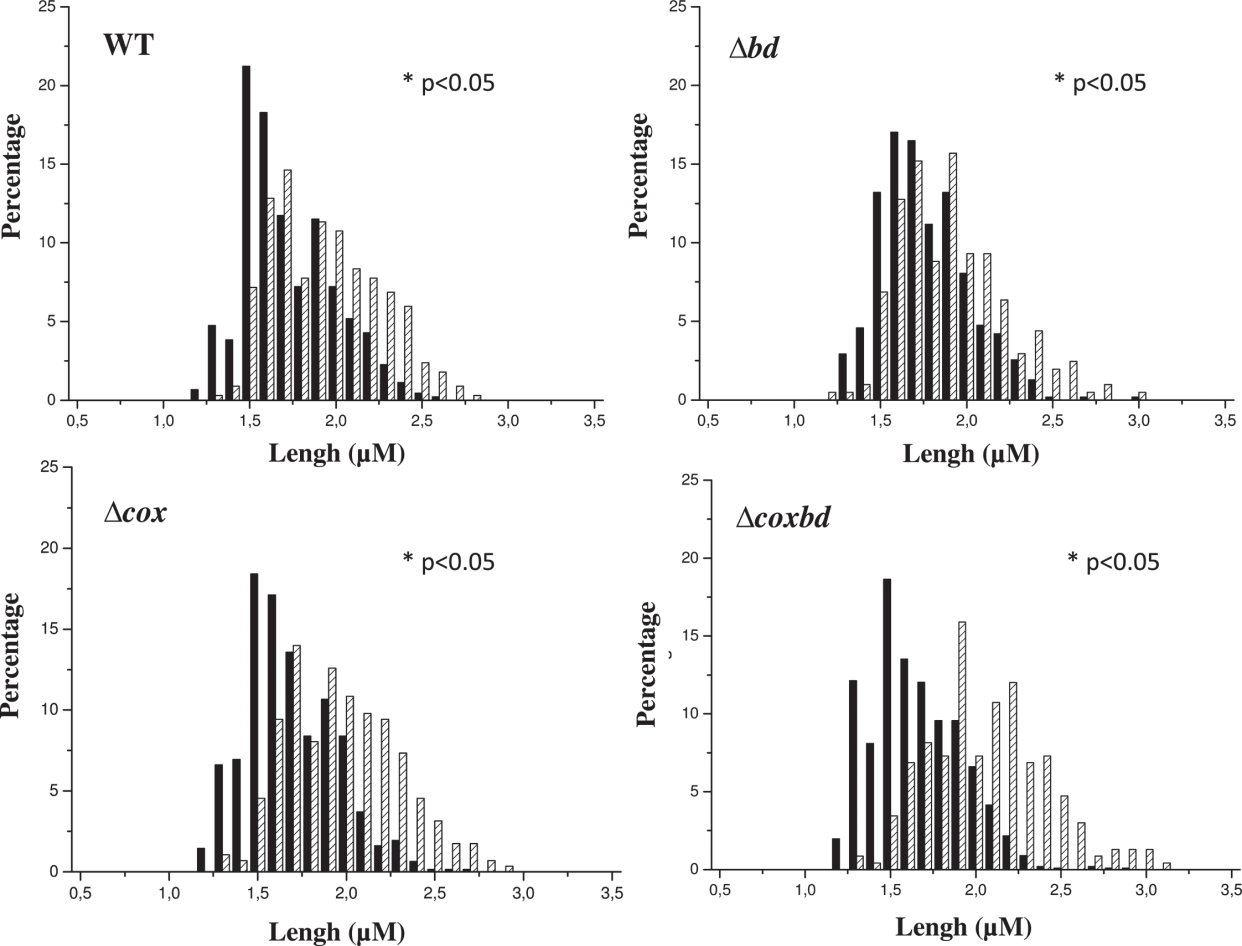
Variability in the cell length of the various *Dv*H strains. Comparison of the distribution of the cell length in cultures under anaerobic conditions (black bars) and with constant 0.02% O_2_ gas mixture sparging (striped bars). The average cell length was determined from two independent cultures (>200 cells were counted in each experiment). Statistical analysis using One-way ANOVA were performed to reveal significant differences between distributions (p<0.05), which were mentioned by an asterisk.

It has been observed that some *Desulfovibrio* strains developed atypically elongated cells when growing in the presence of oxygen [25]. A recent analysis at the single-cell level showed that when *Dv*H was exposed to low oxygen concentration (up to 0.05%), cells elongated (Fievet, unpublished). In order to determine whether the presence or the absence of the membrane bound oxygen reductases would affect this elongation phenomenon, morphology of the cells was studied by microscopy after 24 h of growth with either constant 0.02% O_2_ gas mixture sparging or anaerobic conditions. When cultured under anaerobic conditions, the lengths distribution of the wild-type and the three mutant cells were similar, with a median value around 1.6 μm (Fig 2, S1 Table). When cultured with constant 0.02% O_2_ sparging, cells lengths were greater for all strains. However, variations in lengths distribution differed from strain to strain: while the median length of wild-type and *Δbd* cells were slightly shifted to larger values, + 0.23 μm and + 0.12 μm, respectively, compared to the values measured under anaerobic conditions, the shift of the median lengths of the *Δcox* and *Δcoxbd* cells was more pronounced, + 0.3 μm and + 0.41 μm, respectively (Fig 2, S1 Table). These data show that, when cells were cultured with constant 0.02% O_2_ sparging, the median as well as the extreme lengths values of the cells increased and that the absence of the cytochrome c oxidase had a more pronounced effect on this morphology parameter than that of the bd-quinol oxidase.

### Substrate consumption analysis

**Fig 3:**
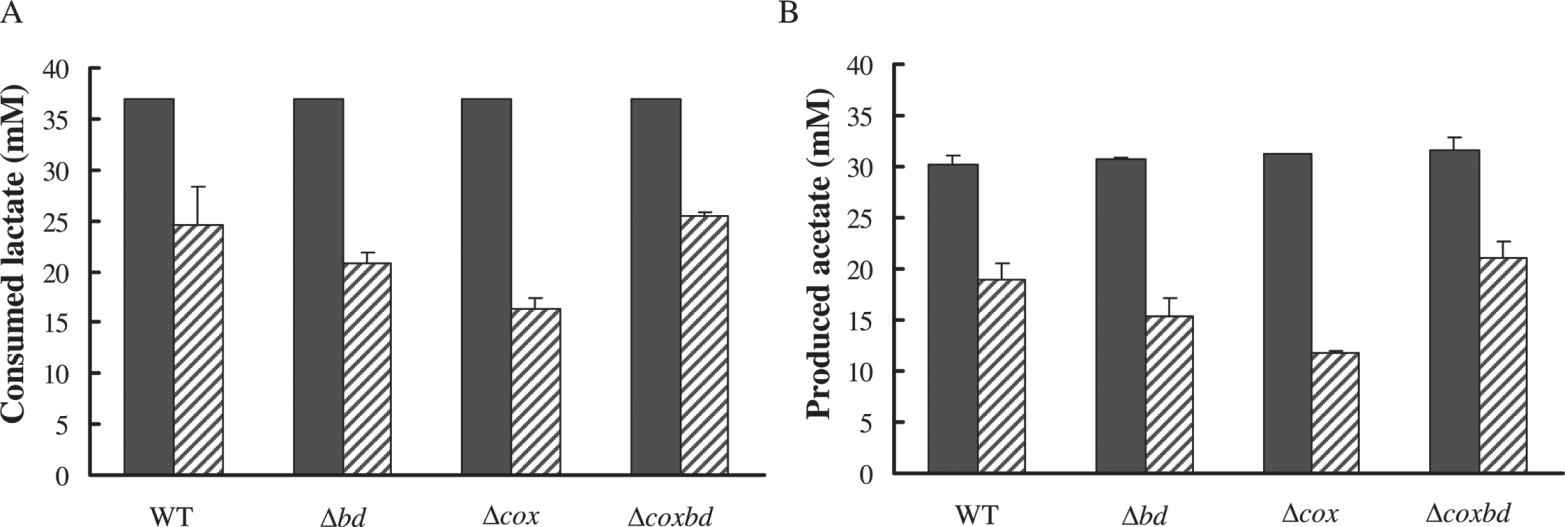
Lactate consumption and acetate production in the *Dv*H strains. Quantification of consumed lactate (A) and produced acetate (B) after 40h growth under anaerobic conditions (black bars) or with constant 0.02% O_2_ gas mixture sparging (striped bars) in medium C (with 37mM of lactate as initial concentration).

**Fig 4:**
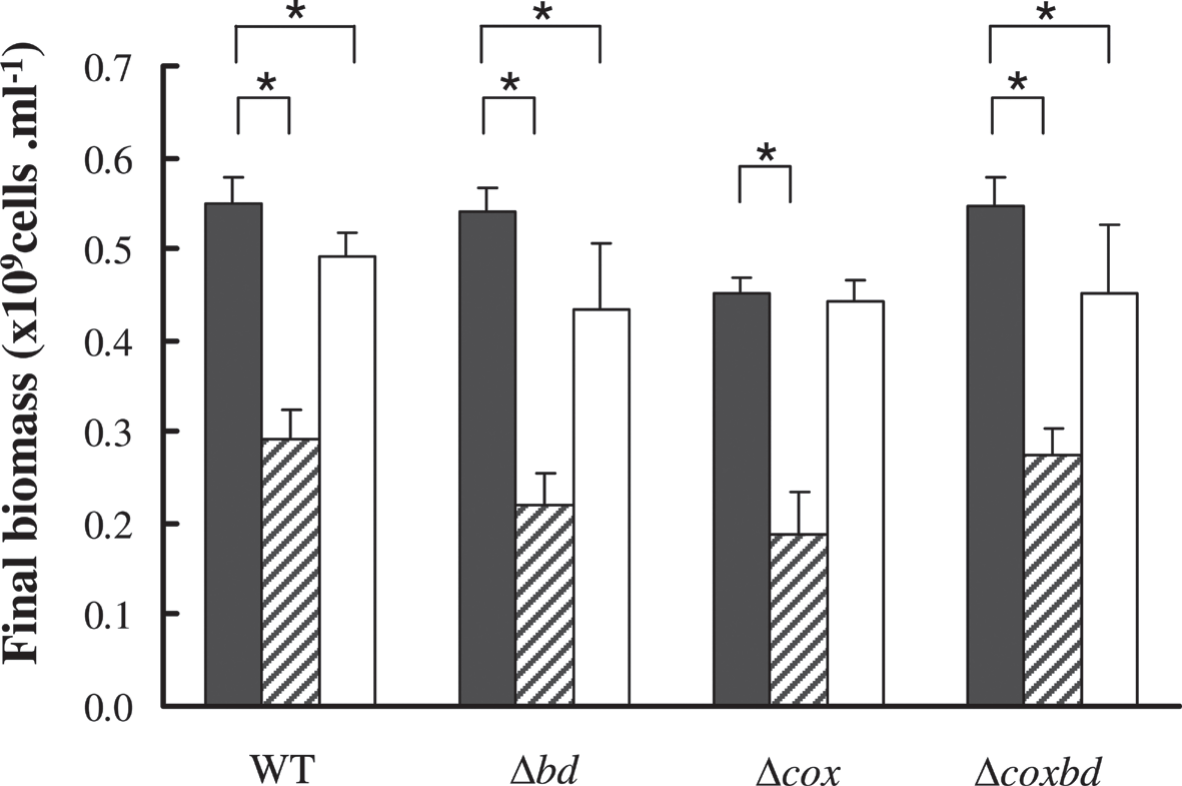
Biomass formation under various culturing conditions. Final biomass for the four *Dv*H strains cultured under anaerobic conditions only (black bars), with constant 0.02% O_2_ gas mixture sparging (striped bars) and after growth resumption (open bars). Data are mean values of five independent experiments + SD. Statistical analysis using t-test was used to compare means. Significant differences (p< 0.05) are mentioned by an asterisk.

**Fig 5:**
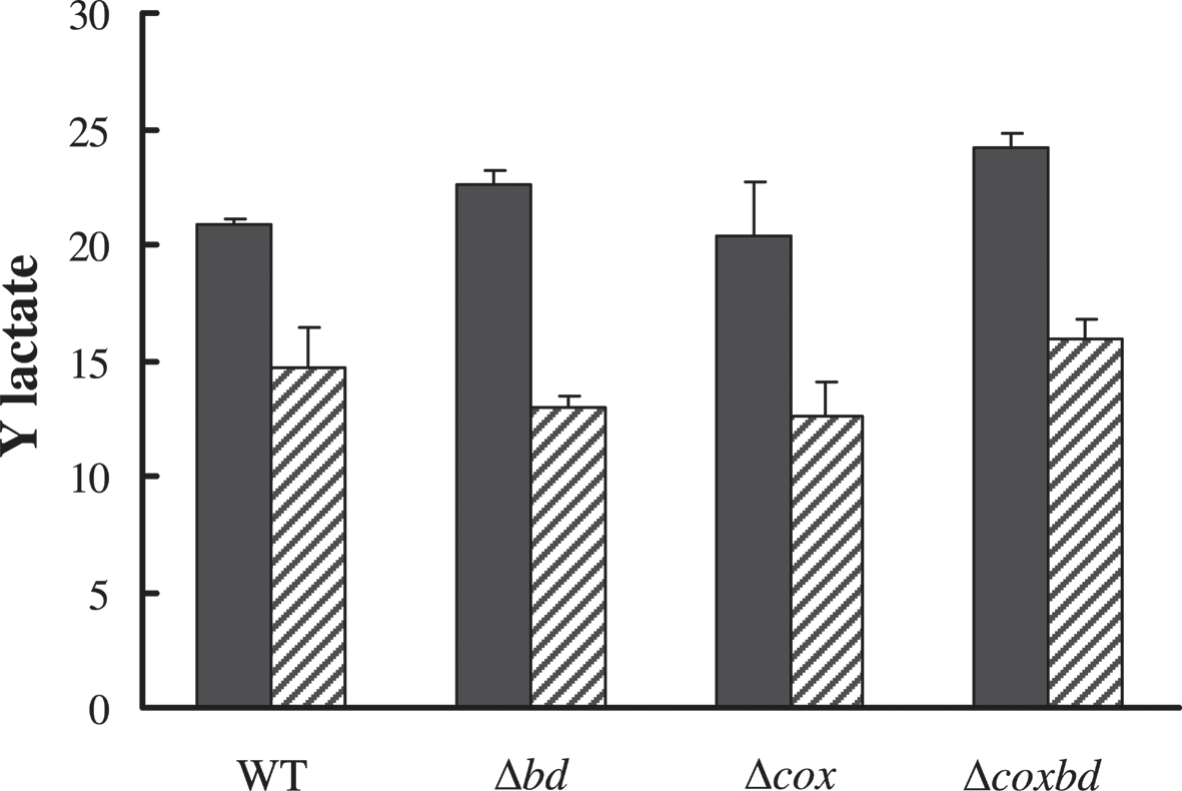
Molar growth yield for the four strains under various growth conditions. Molar growth yield on lactate for the wild-type and deletion mutant strains cultured under anaerobic conditions (black bars) and with constant 0.02% O_2_ gas mixture sparging (striped bars). Molar growth yields are expressed in 10^12^ cells/mole of substrate.

In order to determine whether the growth arrest in the presence of 0.02% O_2_ was due to a complete consumption of substrate (i.e. lactate), lactate and acetate were quantified in the cultures when cells stopped to grow (after ~ 40 h) (Fig 1). Fig 3 shows that under anaerobic conditions, lactate (initial concentration 37 mM) was completely consumed and, accordingly, acetate was produced with an acetate/lactate stoichiometric ratio of about 0.83 for all strains, in agreement with a part of acetate used for biosynthesis. In the cultures with constant 0.02% O_2_ gas mixture sparging, when growth was stopped, 22.2 ± 4.6 mM lactate was consumed in the wild-type culture and 12.6 ± 1.3, 17.8± 1.3 and 23.4± 0.3 mM lactate were consumed in the *Δcox*, *Δbd* and *Δcoxbd* cultures, respectively and accordingly, less acetate was formed (Fig 3B). However, the acetate/lactate stoichiometric ratio was found slightly lower with an average value of 0.74. These data show that the growth arrest, when cultures were constantly sparged with 0.02% O_2_, was not the effect of a complete consumption of the energy substrate by the cells. Interestingly, when conditions were then switched to anaerobic by bubbling the cultures with 100% N_2_ for 15 min to remove oxygen and kept afterwards anaerobic, all strains were able to resume growth and, at the end of the growth, all lactate was consumed (data not shown) in the four strains; however, the final biomasses never reached the value obtained when cells were cultured under anaerobic conditions only, except for the *Δcox* deletion mutant (Fig 4). When cultured with constant 0.02% O_2_ sparging, the molar growth yields on lactate (Y_lactate_) were 70%, 57%, 62% and 65% lower for the wild-type, *Δbd*, *Δcox* and *Δcoxbd* strains, respectively, than when cultured under anaerobic conditions (Fig 5). The single deletion mutant *Δbd* and *Δcox* strains exhibited the lowest values while, surprisingly, Y_lactate_ of the double deletion mutant was greater than those of the single deletion mutants and similar to that of the wild-type strain (Fig 5).

### Quantification of number of cells able to divide after oxygen exposure

When cells were exposed to a constant 0.02% O_2_ sparging, they stopped to grow (Fig 1). In order to quantify the number of cells that were able to divide again when conditions were then switched to anaerobic, time-lapse microscopy on both the wild-type and deletion mutant strains was performed. Table 2 shows that in the case of the wild-type and the *Δcoxbd* strains, 100% of cells were able to divide when conditions were switched to anaerobic, while 1% and 2.4% of *Δbd* and *Δcox* cells, respectively, were unable to restart division. The oxidative conditions induced by the constant 0.02% O_2_ sparging did not thus have any significant effect on the capabilities of the wild-type and the *Δcoxbd* strains to resume division when conditions were switched to anaerobic and had only a weak effect on the *Δbd* and *Δcox* deletion mutant strains for which a low percentage of cells were found unable to resume division in anaerobic conditions.

**Table 2:** Quantification of cells able to divide and average division parameters of the wild-type and deletion mutant strains after oxygen exposure.

****Genotype****	****Cells unable to divide (%)****	****First division recovery time (h)****	****Second doubling time (h)****
WT	0	5.29 ± 0.25 ^a^	1.86 ± 0.12 ^b^ ^,^ ^c^
*Δbd*	1	6 ± 0.43 ^a^	2.49 ± 0.47 ^b^
*Δcox*	2.4	5.27 ± 0.91	2.42 ± 0.19 ^c^
*Δcoxbd*	0	5.26 ± 0.97	2.06 ± 0.03

Strains were cultured with constant 0.02% O_2_ gas mixture sparging followed by a switch to anaerobic condition. Data are mean values of three independent experiments ± SD. Statistical analysis using t-test was used to compared means.

^a, b, c^: Significant differences.

**Fig 6:**
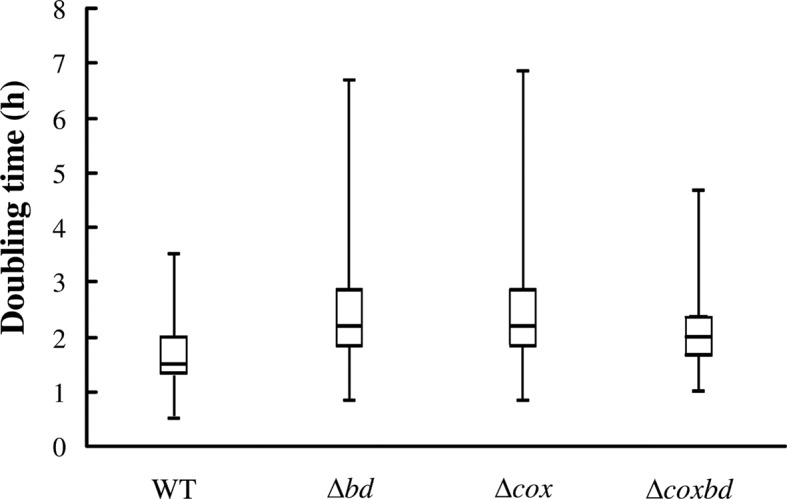
Variability of the second doubling time for the four strains after oxygen exposure. Box and whiskers plot representing the variability in the distribution of the second doubling time of the various *Dv*H strains cultured with constant 0.02% O_2_ gas mixture sparging followed by a switch to anaerobic condition. These distribution patterns arose from at least 2 independent cultures (> 200 cells each).

The average values of the first division recovery time (time to recover a complete division event), calculated on at least 200 cells, were 6 hours for the *Δbd* mutant and about 5.3 hours for the three others strains (Table 2). When the same experimental protocol was performed on cells cultured under anaerobic conditions only (without any exposure to oxygen), the time to recover a complete division event was about 3 h for all strains (data not shown). The second doubling time (time to finish the second division event) was about 2.5 hours in average for the two single deletion mutants (*Δbd* and *Δcox*) and shorter for the *Δcoxbd* (2.1 hours) and the wild-type (1.9 hours) (Table 2). The distribution pattern showed that second doubling times for the single deletion mutant *Δbd* and *Δcox* strains were more scattered than that of the wild-type, with a significant proportion of cells exhibiting higher median, third quartile and maximum values. The distribution pattern of double deletion mutant *Δcoxbd* appeared closer to that of the wild-type strain, with less scattered values (Fig 6). These data show that even if cells were able to divide again when conditions were switched to anaerobic, it took longer to complete a division event when cells were previously exposed to constant 0.02% O_2_ sparging, the *Δbd* strain being the most affected. In addition the absence of either one of the membrane-bound oxygen reductase had a pronounced effect on the following division event, which was longer than for the wild-type strain.

### PFOR activity on the various strains

**Fig 7:**
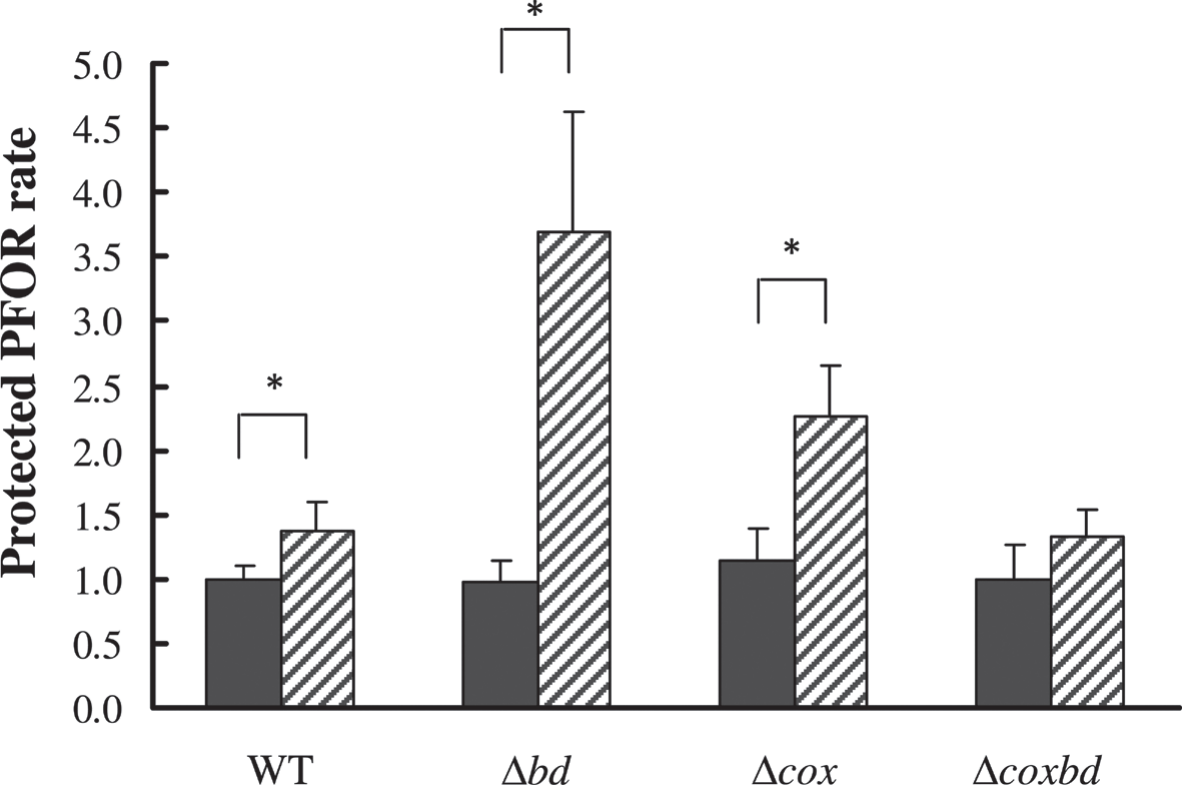
Protected PFOR rate in the four strains. Quantification of the rate of protected PFOR during the exponential growth phase under anaerobic (black bars) and with constant 0.02% O_2_ gas mixture sparging (striped bars). Statistical analysis using Mann and Whitney test was used to compare means. Significant differences (p< 0.05) are indicated by an asterisk. All values are related to the PFOR protected rate in the wild-type strain under anaerobic conditions (value arbitrary set to 1).

The above data point out that when *Dv*H was cultured with constant 0.02% O_2_ gas mixture sparging, it was able to divide more than two times, then stopped growing and, when conditions were switched to anaerobic, resumed dividing. The pyruvate-ferredoxin oxidoreductase (PFOR) has been shown to be able to reversibly switch from a fully active oxidative-sensitive state (active state) to an inactive oxidative-insensitive state (protected state) when cells encounter oxidative conditions by means of an autoprotective thiol-redox mechanism [24,50]. Because this enzyme plays a crucial role in the lactate metabolism, effect of constant 0.02% O_2_ sparging on the PFOR state was studied. Quantification of the PFOR activity in cells without and with addition of a chemical reducing agent (i. e. dithioerythritol) stands for the active *versus* protected states of the enzyme [24]. The protected PFOR rate was determined for each strain when cells were cultured under both anaerobic conditions and with constant 0.02% O_2_ sparging (Fig 7). While under anaerobic conditions, the protected PFOR rate was found similar in all strains (the rate being arbitrary set to 1 for the wild-type strain), when cells were cultured with constant 0.02% O_2_ sparging, this rate was found slightly higher in the wild-type strain than under anaerobic conditions and much higher for the two single deletion mutant strains, the *Δbd* strain exhibiting the largest one. On the contrary, the double deletion mutant exhibited a similar rate as the wild-type strain. It should be noted that no variation of the amount of *por* transcripts induced by the oxidative conditions caused by the constant 0.02% O_2_ sparging was observed in any strain (data not shown). These data show that under oxidative conditions, the PFOR protected rate depended on the presence of the membrane bound oxygen reductases, their absences, and specially that of the bd oxidase, leading to a larger part of the PFOR in the protected form. In the case of the double mutant, one or several other mechanisms, still unknown, would balance the absence of the two oxygen reductases.

## Discussion

To our knowledge, only one study [32] has reported the growth of *Desulfovibrio* species with constant oxygen sparging with more than a doubling of the initial population. In this report, we successfully grew *Dv*H in Hungate tubes with lactate as carbon and energy sources and sulfate as electron acceptor, with constant 0.02% O_2_ gas mixture sparging, corresponding to a constant dissolved oxygen concentration of 0.23μM. It should be noted that no formation of bacterial aggregates which would permit cells to create anaerobic micro-niches as in *Desulfovibrio oxyclinae* [34] was observed. Under these conditions, *Dv*H was able to divide more than two times but growth parameters were affected, i.e. lower growth rate and final biomass than under anaerobic conditions. An important point is that cells stopped to grow despite lactate was not completely consumed. This growth arrest was not due to cells death as time-lapse microscopy revealed that all cells were able to re-start division when the conditions were switched to anaerobic. This high survival rate under oxidative conditions is in agreement with the high aerotolerance of *Dv*H that was previously pointed out [26,44,52]. Interestingly, growth resumed if conditions were switched to anaerobic, with the recovery of lactate consumption until exhaustion but the final biomass did not reach the same value as when cells were grown under completely anaerobic conditions. The molar growth yield on lactate was found lower when cells were cultured with constant 0.02% O_2_ gas mixture sparging than under anaerobic conditions, indicating that a part of the energy gained from the substrate oxidation is derived toward cells protection/repairing against oxidative conditions rather than biosynthesis. A trade-off should exist between growth and defense investment, permitting bacteria to redirect energy for growth to repairing or detoxifying systems.

These observations directed our attention to the potential involvement of the two membrane-bound oxygen reductases, the bd-quinol oxidase and the cytochrome cc(o/b)o_3_ oxidase, in the growth under the continuous presence of low oxygen concentration. The study of the single deletion mutant strains of the genes encoding these oxygen reductases revealed that the absence of either of the two membrane-bound oxygen reductases led to both lower growth rates and lower final biomasses than those observed for the wild-type strain when cultured with constant 0.02% O_2_ sparging. As for the wild-type strain, the molar growth yields on lactate of the deletion mutant strains are lower with constant 0.02% O_2_ sparging, compared to anaerobic conditions, with a larger difference for the single *Δbd*, and *Δcox* deletion mutant strains. It suggests that the energy diverted to repairing/detoxifying systems is higher in the single deletion *Δbd* and, to a lesser extent, *Δcox* mutant strains, indicating that the membrane-bound oxygen reductases, and especially the bd-quinol oxidase, play an important role in maintaining cells healthy under oxidative conditions. They could be involved through their capability to both detoxify oxygen by reducing it to water and contribute to the generation of a proton motive force inherent in their mechanistic activities [53–54]. The prevailing role of the bd-quinol oxidase is in agreement with its function in aerobic organisms where it significantly contributes to respiration of O_2_ under microaerobic conditions, when oxygen availability is limited [55], which is compatible with its high affinity for oxygen [56–57]. In addition, it has to be related with our previous results that have shown a higher affinity for oxygen of the bd-quinol oxidase (Km 300 nM) than that of the cytochrome cc(o/b)o_3_ oxidase (Km 620 nM) as well as a greater abundance of the former enzyme in *Dv*H cells [26]. Contrary to expectation, the double deletion mutant does not appear more affected than the single deletion mutants when cultured under constant 0.02% O_2_ sparging, and even, on several aspects (final biomass, Y_lactate_, second division time, PFOR protected rate), has a behaviour quite similar as that of the wild type. While the viability of double deletion mutant after exposure to air or to 0.1% O_2_ for more than 8 hours was lower than that of the single deletion mutant strains [26], the present data suggests that under these specific oxidative conditions, the absence of the two membrane-bound oxygen reductases has an beneficial effect when compared to the absence of only one. The mechanism that would balance the absence of the two membrane-bound reductases is however still unknown and studies are needed to get further insights.

The fact that *Dv*H, when cultured with constant 0.02% O_2_ gas mixture sparging, is able to grow for more than two generations, then stops growing but keeps its ability to divide and to resume growing when conditions are switched to anaerobic, suggests that it is able of active dormancy as originally proposed by Le Gall and Xavier [58]. The idea was that when exposed to oxygen, cells were not killed but remained dormant with the maintenance of low-level cell activities [58–59]. Ability of anaerobes to enter in active dormancy should be considered as an adaptation strategy used to cope with temporary exposure to O_2_. The maintenance of cells activity under oxidative conditions enables them to rapidly resume growth on re-entering an anaerobic habitat. Culturing *Dv*H with 0.02% O_2_ sparging would induce the cells to enter active dormancy state after several hours. The longer time to recover a complete division event when conditions are then switched to anaerobic than when *Dv*H is continuously cultured under anaerobic conditions (about 5.3 h *versus* 3 h) as observed by microscopy, could be linked to the need of a metabolic reprogramming to get out active dormancy and to resume dividing. In the case of the *Δbd* deletion mutant strain, the absence of the bd-quinol oxidase could have induced more oxidative damages that have to be fixed before the cells can divide again, which result in a longer time to recover a division event (6 h versus 5.3 h).

The mechanism by which cells are able to enter active dormancy is still unknown but the Pyruvate-Ferredoxin OxidoReductase (PFOR) should play an important role through its autoprotective disulfide redox-switch mechanism [24]. This mechanism allows the enzyme to reversibly switch from a fully active oxidative-sensitive state to an inactive oxidative-insensitive state when cells encounter oxidative conditions [24,50]. Protecting PFOR from oxidative damages and restoring a high rate of pyruvate oxidation by switching PFOR to its fully active form without *de novo* synthesis could be a key stage for rapid growth resumption. The protected state of the PFOR can be thus considered as an hallmark of the active dormancy state. Our data show that when *Dv*H is cultured under constant 0.02% O_2_ sparging, a part of the PFOR is switched from active to protected states, inducing a decrease of the pyruvate oxidation activity in the cells. This decrease would lead cells to enter active dormancy, resulting in a growth arrest. When conditions become less oxidative, PFOR is switched to active state, restoring a high pyruvate oxidation activity, allowing cells to get out active dormancy state and to resume growth. The membrane-bound oxygen reductase, and especially the bd-quinol oxidase, seem to play an important role in the protected/active states PFOR switch. In *Azotobacter vinelandii*, the bd-quinol oxidase has be shown to play an important role in protecting the O_2_ labile nitrogenase activity by maintaining very low O_2_ concentration [60] In the same way, one can propose that the bd-quinol oxidase in *Dv*H would play a crucial role in maintaining inside the cell very low oxygen concentration and thus reducing conditions that permit PFOR to stay in its active state. When O_2_-reduction activity is not efficient enough, increased oxidative conditions inside the cell would trigger it to enter active dormancy.

Characterization of the active dormancy state constitutes the next challenge to get a better understanding of the aerotolerance capabilities of anaerobic microorganisms.

## Supporting Information

S1 Fig Quantification of *cox* and *bd* genes transcripts by qRT-PCR. Transcript level of the bd-quinol oxidase encoding gene (*bd* gene) (A) and the cytochrome c oxydase encoding gene (*cox* genes) (B) in WT and deletion mutants in anaerobiosis (black bars) or continuously exposed to 0.02% O_2_ sparging (striped bars). Data are mean values of two independent experiments +SD.
(DOC)

S1 Table Variability in the cell length of the various *Dv*H strains. Variability in the cell length of the various *Dv*H strains. Values of cell length (μm) in cultures under anaerobic conditions and with a constant 0.02% O_2_ gas mixture sparging for 24 hours for each strain. The quartiles values (Q1, Q3) indicated in the table come from two independent cultures (>200 cells were counted in each experiment).
(DOC)
