# Supplementary material for: Growth of the Obligate Anaerobe Desulfovibrio vulgaris Hildenborough under Continuous Low Oxygen Concentration Sparging: Impact of the Membrane-Bound Oxygen Reductases
Source: PLoS One. 2015 Apr 2;10(4):e0123455. doi: 10.1371/journal.pone.0123455 (PMC4383621; doi:10.1371/journal.pone.0123455)
Supplement: S1 Fig — Transcript level of the bd-quinol oxidase encoding gene (bd gene) (A) and the cytochrome c oxydase encoding gene (cox genes) (B) in WT and deletion mutants in anaerobiosis (black bars) or continuously exposed to 0.02% O2 sparging (striped bars). Data are mean values of two independent experiments +SD. (DOC) [file pone.0123455.s001.doc]

**Figure S1:** Transcript level of the bd-quinol oxidase encoding gene (*bd* gene) (A) and the cytochrome c oxydase encoding gene (*cox* genes) (B) in WT and deletion mutants in anaerobiosis (black bars) or continuously exposed to 0.02 % O_2_ sparging (striped bars)**.** Data are mean values of two independent experiments +/- SD.
