# Supplementary material for: Growth of the Obligate Anaerobe Desulfovibrio vulgaris Hildenborough under Continuous Low Oxygen Concentration Sparging: Impact of the Membrane-Bound Oxygen Reductases
Source: PLoS One. 2015 Apr 2;10(4):e0123455. doi: 10.1371/journal.pone.0123455 (PMC4383621; doi:10.1371/journal.pone.0123455)
Supplement: S1 Table — Variability in the cell length of the various DvH strains. Values of cell length (μm) in cultures under anaerobic conditions and with a constant 0.02% O2 gas mixture sparging for 24 hours for each strain. The quartiles values (Q1, Q3) indicated in the table come from two independent cultures (>200 cells were counted in each experiment). (DOC) [file pone.0123455.s002.doc]

**Table S1:** Variability in the cell length of the various *Dv*H strains. Values of cell length (µm) in cultures under anaerobic conditions and with a constant 0.02% O_2_ gas mixture sparging for 24 hours for each strain. The quartiles values (Q1, Q3) indicated in the table come from two independent cultures (>200 cells were counted in each experiment).

.
